# Genetic Predictors of Paxlovid Treatment Response: The Role of IFNAR2, OAS1, OAS3, and ACE2 in COVID-19 Clinical Course

**DOI:** 10.3390/jpm15040156

**Published:** 2025-04-17

**Authors:** Mykhailo Buchynskyi, Iryna Kamyshna, Iryna Halabitska, Pavlo Petakh, Valentyn Oksenych, Oleksandr Kamyshnyi

**Affiliations:** 1 Department of Microbiology, Virology, and Immunology, I. Horbachevsky Ternopil National Medical University, 46001 Ternopil, Ukraine; kamyshnyi_om@tdmu.edu.ua; 2 Department of Medical Rehabilitation, I. Horbachevsky Ternopil National Medical University, 46001 Ternopil, Ukraine; kamyshna_ii@tdmu.edu.ua; 3 Department of Therapy and Family Medicine, I. Horbachevsky Ternopil National Medical University, Voli Square, 1, 46001 Ternopil, Ukraine; halabitska@tdmu.edu.ua; 4 Department of Biochemistry and Pharmacology, Uzhhorod National University, 88000 Uzhhorod, Ukraine; pavlo.petakh@uzhnu.edu.ua; 5 Broegelmann Research Laboratory, Department of Clinical Science, University of Bergen, 5020 Bergen, Norway

**Keywords:** COVID-19, Paxlovid, nirmatrelvir, IFNAR2, OAS1, OAS3, ACE2

## Abstract

**Background:** This study investigated the role of genetic polymorphisms in IFNAR2, OAS1, OAS3, and ACE2 as predictors of Paxlovid treatment response, specifically examining their influence on the clinical course and laboratory parameters of COVID-19 patients. **Methods:** We analyzed the impact of polymorphisms in genes associated with the interferon pathway (IFNAR2 rs2236757), antiviral response (OAS1 rs10774671, OAS3 rs10735079), and viral entry (ACE2 rs2074192) in individuals treated with Paxlovid. **Results:** Our findings suggest that genetic variations in these genes may modulate the immune response and coagulation pathways in the context of Paxlovid treatment during COVID-19 infection. Specifically, the IFNAR2 rs2236757 G allele was associated with alterations in inflammatory and coagulation markers, while polymorphisms in OAS1 and OAS3 influenced coagulation parameters. Furthermore, specific genotypes were linked to changes in clinical parameters such as oxygen saturation, leukocyte count, and liver function markers in Paxlovid-treated patients. **Conclusions:** These results highlight the potential of considering genetic factors in understanding individual responses to COVID-19 treatment with Paxlovid and informing future personalized approaches.

## 1. Introduction

Since the onset of the severe acute respiratory syndrome coronavirus 2 (SARS-CoV-2) pandemic, a wide range of antiviral therapeutic strategies have been investigated to combat COVID-19 [1,2,3,4].

Paxlovid, a combination therapy comprising nirmatrelvir and ritonavir, has garnered significant attention as a potential treatment option [5,6,7,8]. Nirmatrelvir, a specific inhibitor targeting the SARS-CoV-2 main protease, is synergistically enhanced by ritonavir, a cytochrome P450 3A4 inhibitor [9,10,11,12,13]. Several studies have demonstrated the efficacy of Paxlovid in reducing COVID-19-related mortality and hospitalization rates [14,15,16,17]. In particular, a randomized controlled trial reported an 89% reduction in the risk of hospitalization and death within 28 days when Paxlovid was administered within 3 days of symptom onset [18].

The limited availability of data on adverse events associated with the nirmatrelvir-ritonavir combination hinders a comprehensive understanding of its safety profile [19,20,21,22,23]. Clinical studies have documented dysgeusia, characterized by alterations in taste perception, and diarrhea as common side effects [15,24,25,26,27]. A comprehensive analysis by Li et al. [28] further elucidated the spectrum of adverse events associated with nirmatrelvir-ritonavir administration. The most commonly reported adverse events were mild to moderate in severity and included dysgeusia, diarrhea, nausea, headache, pyrexia, vomiting, and malaise [29,30,31].

Differences in drug absorption, distribution, metabolism, and excretion can lead to varying drug concentrations in different individuals [32,33,34]. For instance, genetic polymorphisms in drug-metabolizing enzymes can affect the rate at which a drug is metabolized, influencing its efficacy and potential side effects [34,35,36,37]. Drugs primarily metabolized by a single enzyme and with a broad therapeutic index may exhibit significant pharmacokinetic variability due to pharmacogenetic variants [38,39,40]. However, given their wide therapeutic window, these genetic differences may not necessarily translate into clinically relevant drug efficacy or toxicity variations [41,42,43,44]. For instance, drug interactions or underlying diseases that inhibit one metabolic pathway and genetic variations impairing a second pathway can contribute to atypical drug responses [45,46,47]. Certain medications, originally prescribed for other conditions, exhibit pleiotropic effects that contribute to their efficacy in the treatment of COVID-19 [48,49,50,51]. These drugs, beyond their primary therapeutic action, engage additional mechanisms that influence the pathogenesis of COVID-19, thereby enhancing clinical outcomes in affected patients. [52,53,54]. Evidence suggests that certain medications, typically used for other conditions, may reduce the severity of COVID-19 outcomes in individuals with metabolic disorders, such as type 2 diabetes, by mitigating complications related to hyperglycemia [55,56,57]. The repurposing of existing drugs for diverse diseases is highly valuable, as it utilizes established pharmacological insights to expedite the discovery of effective treatments [58,59,60,61].

Genetic polymorphisms have been implicated in both the susceptibility to and severity of COVID-19, affecting various biological pathways relevant to the disease [62,63,64,65]. The angiotensin-converting enzyme 2 (*ACE2*) receptor is the primary cellular receptor for SARS-CoV-2 entry [62,66,67]. The intronic variant rs2074192 has been implicated in changes to the secondary structure of *ACE2* mRNA, potentially disrupting the equilibrium between *ACE2* transcription and translation [64,68]. This imbalance may consequently affect the binding affinity of SARS-CoV-2 to angiotensin receptors [63,69,70]. Notably, the *rs2074192* polymorphism in the *ACE2* gene has been implicated in COVID-19 severity, particularly in adult populations [64,71,72].

Furthermore, genetic variations affecting immune response pathways, such as those involving interferon and cytokine signaling, have been associated with differential susceptibility to infection and disease severity [73,74,75,76]. Variations in interferon genes or their receptors have been linked to increased susceptibility or more severe clinical outcomes [65,77,78]. The *IFNAR2 rs2236757* variant, in particular, has been strongly associated with increased disease severity [65,79,80]. Furthermore, polymorphisms in antiviral 2′,5′-oligoadenylate synthetase (*OAS*) enzymes, which are essential for the immune response against SARS-CoV-2, have also been implicated in COVID-19 severity [65,81,82]. SNPs such as *rs10774671* in *OAS1* [83] and *rs10735079* in *OAS3* [65] may be associated with more severe clinical outcomes following COVID-19 infection [84,85].

As Paxlovid has become a standard treatment for COVID-19, further research is essential to fully understand its impact on clinical and laboratory parameters [86,87,88]. The presence of specific single nucleotide polymorphisms and their potential effects on treatment outcomes adds to the complexity of managing COVID-19 patients [89,90]. These genetic polymorphisms have been studied as predictors of COVID-19 severity. Considering that these patients are indicated for treatment against COVID-19, investigating the combined effect of these polymorphisms and treatment response is crucial. This study examines their association with treatment and clinical and laboratory parameters for the first time.

## 2. Materials and Methods

### 2.1. Sample Collection

This study included 72 adults of European ancestry (Ukrainian ethnicity) aged 23 to 86 years who tested positive for SARS-CoV-2 and were subsequently hospitalized between October 2022 and May 2023. Confirmation of SARS-CoV-2 infection was achieved through real-time polymerase chain reaction (RT-PCR) analysis of nasopharyngeal swab samples. Study participants were enrolled from Ternopil City Community Hospital No. 1. Informed consent was obtained prior to the collection of blood samples, which were subsequently stored at −80 °C for subsequent analysis. All research procedures were conducted in strict adherence to the ethical principles outlined in the Declaration of Helsinki. Prior to the commencement of any study activities, the research protocol (No. 74, dated 13 October 2023) underwent rigorous ethical review and received formal approval from the Ethics Committee of the I. Horbachevsky Ternopil National Medical University.

Participants were included if they had a confirmed COVID-19 diagnosis requiring hospitalization, no history of chronic diseases, and no antibiotic or probiotic use within the past three months.

All study participants adhered to the standard treatment protocol for COVID-19 as outlined by national guidelines. This regimen included symptomatic relief: antipyretic therapy with paracetamol or ibuprofen; respiratory support: mucolytic and expectorant agents, such as Ambroxol, and non-invasive oxygen therapy as needed; thromboembolic prophylaxis: anticoagulant therapy with low-molecular-weight heparins, like enoxaparin, at a dose of 40 mg or 4000 IU anti-Xa; antimicrobial therapy: prophylactic antimicrobial treatment for potential co-infections, consisting of amoxicillin/clavulanate combined with macrolides (azithromycin or clarithromycin) or cephalosporins of the second or third generation; and immunomodulation: corticosteroid therapy with intravenous dexamethasone at a dose of 0.15 mg/kg daily (8–16 mg) for 7–10 days. The use of corticosteroids and antibiotics did not differ between the groups.

Twenty-three out of seventy-three patients received nirmatrelvir–ritonavir (Paxlovid) in accordance with Food and Drug Administration (FDA) recommendations, consisting of an oral dose of 300/100 mg twice daily for 5 days [91].

### 2.2. Laboratory and Clinical Data

A comprehensive laboratory workup was performed, including assessment of oxygen saturation, complete blood count (CBC) with differential, erythrocyte sedimentation rate (ESR), coagulation profile (platelet count, hematocrit, INR, PT), quick pro-thrombin time (QTP), activated partial thromboplastin time (APTT), fibrinogen, total bilirubin, alanine aminotransferase (ALT), aspartate aminotransferase (AST), serum creatinine, gamma-glutamyl transferase (GGT), total protein, albumin, alkaline phosphatase (ALP), C-reactive protein (CRP), and blood glucose.

### 2.3. Identifying Genetic Polymorphisms

Venous blood samples were collected, and genomic DNA was extracted using a commercial kit, the Thermo Scientific™ GeneJET™ Whole Blood Genomic DNA Purification Mini Kit Cat. No. K0781. Polymorphisms in the *ACE2 rs2074192*, *IFNAR2 rs2236757*, *OAS1 rs10774671*, and *OAS3 rs10735079* genes were analyzed using real-time PCR. PCR amplification and melting curve analysis were performed using TaqMan assays under optimized conditions. The CFX96™ Real-Time PCR Basic Software was utilized for genotyping analysis based on the melting curve.

### 2.4. Statistical Analysis

Descriptive statistics were utilized to delineate the demographic characteristics and clinical outcomes of the study population. Due to non-normal data distribution, non-parametric tests were employed for comparisons. The Mann–Whitney U test, Kruskal–Wallis test, Dunn’s multiple comparison test, and Wilcoxon matched pairs test were utilized for appropriate comparisons.

Repeated measures ANOVA was employed to investigate the influence of specific factors on a continuous variable. Post hoc comparisons with Bonferroni correction were subsequently conducted to identify significant differences between groups. In instances where the normality assumption was not sufficiently met, data are displayed as medians and interquartile range (IQR).

A two-tailed alpha level of 0.05 was used to establish statistical significance. Analyses were conducted using GraphPad Prism (version 8.4.3) and IBM SPSS Statistics (version 25).

### 2.5. Power Analysis

To assess the statistical power of our study, we employed the G*power 3.1.9.7 software (https://www.psychologie.hhu.de/arbeitsgruppen/allgemeine-psychologie-und-arbeitspsychologie/gpower(accessed on 2 April 2025). To analyze the impact of genetic variations and Paxlovid treatment on the trajectory of laboratory parameters from hospitalization to discharge, we utilized repeated measures ANOVA. Our study design involved two groups and two measurement points.

Assuming a medium effect size of 0.3, an α-error probability of 0.05, a total sample size of 72, and a correlation among repeated measures of 0.5, our analysis indicates a statistical power of 82%.

## 3. Results

### 3.1. Baseline Patient Parameters

Of the 72 enrolled patients, 23 were assigned to the Paxlovid treatment group (52.17% male; median age of 64 years, IQR 46–71) and 49 to the standard treatment group (63.26% male; median age of 66 years, IQR 51–72). No significant differences were observed between the two groups in terms of demographic characteristics, peripheral oxygen saturation, oxygen therapy requirements, COVID-19 severity, or body mass index, as detailed in Table 1. The median time to hospital discharge was shorter in the Paxlovid treatment group compared with the standard treatment group (9 days, IQR 7–11 vs. 11 days, IQR 9–14, *p* = 0.001).

The genotype distributions for the *ACE2 rs2074192*, *IFNAR2 rs2236757*, *OAS1 rs10774671*, and *OAS3 rs10735079* polymorphisms were found to be in Hardy-Weinberg equilibrium (HWE) in both the Paxlovid and standard treatment groups (*p* > 0.05). Detailed genotype frequency data are presented in Table 2.

### 3.2. Alleles and Clinical Dynamics

To examine the potential interaction between hospital stay (time) and genetic factors (specific SNPs), repeated measures ANOVA was conducted. This analysis assessed both within-subject effects (interaction between time and genetic factors) and between-subject effects (main effects of genetic factors). Effect size was calculated using partial eta-squared (η^2^p) to quantify the influence of each factor on the continuous variable. The results of this analysis are presented in Table 3.

Repeated measures ANOVA revealed a significant interaction effect between time and the presence of the *IFNAR2 rs2236757* G allele on band neutrophil levels (F = 5.051, *p* = 0.028, η^2^p = 0.067), as well as a significant between-subjects effect (F = 7.632, *p* = 0.007, η^2^p = 0.098). Additionally, a significant interaction effect between the presence of the *IFNAR2 rs2236757* G allele and time was observed for platelet count (F = 5.977, *p* = 0.017, η^2^p = 0.079), segmented neutrophils (F = 5.688, *p* = 0.020, η^2^p = 0.075), and fibrinogen levels (F = 5.101, *p* = 0.027, η^2^p = 0.068). Significant between-subjects effects were also found for platelet count (F = 4.468, *p* = 0.038, η^2^p = 0.060) and fibrinogen levels (F = 6.263, *p* = 0.015, η^2^p = 0.082).

Patients carrying the *IFNAR2 rs2236757* A allele exhibited a significant interaction effect between the presence of this allele and time on APTT levels (F = 6.236, *p* = 0.015, η^2^p = 0.082) and albumin levels (F = 6.258, *p* = 0.015, η^2^p = 0.082). Meanwhile, the between-subjects effect was not significant for APTT (F = 0.172, *p* = 0.453, η^2^p = 0.002) or albumin (F = 0.783, *p* = 0.379, η^2^p = 0.011).

Similarly, significant interactions were observed between the presence of the A allele and time for both *OAS3* and *OAS1* polymorphisms on QPT levels (F = 5.380, *p* = 0.023, η^2^p = 0.071 for both *OAS3* and *OAS1*), with non-significant between-subjects effects (F = 0.570, *p* = 0.453, η^2^p = 0.008 for both *OAS3* and *OAS1*). Additionally, significant interactions were found for fibrinogen levels (F = 4.128, *p* = 0.046, η^2^p = 0.056 for *OAS3* and F = 4.452, *p* = 0.038, η^2^p = 0.060 for *OAS1*), with non-significant between-subjects effects (F = 1.951, *p* = 0.167, η^2^p = 0.027 for *OAS3* and F = 2.748, *p* = 0.102, η^2^p = 0.038 for *OAS1*).

Paxlovid treatment did not interact significantly with any laboratory outcomes.

**Figure 1:**
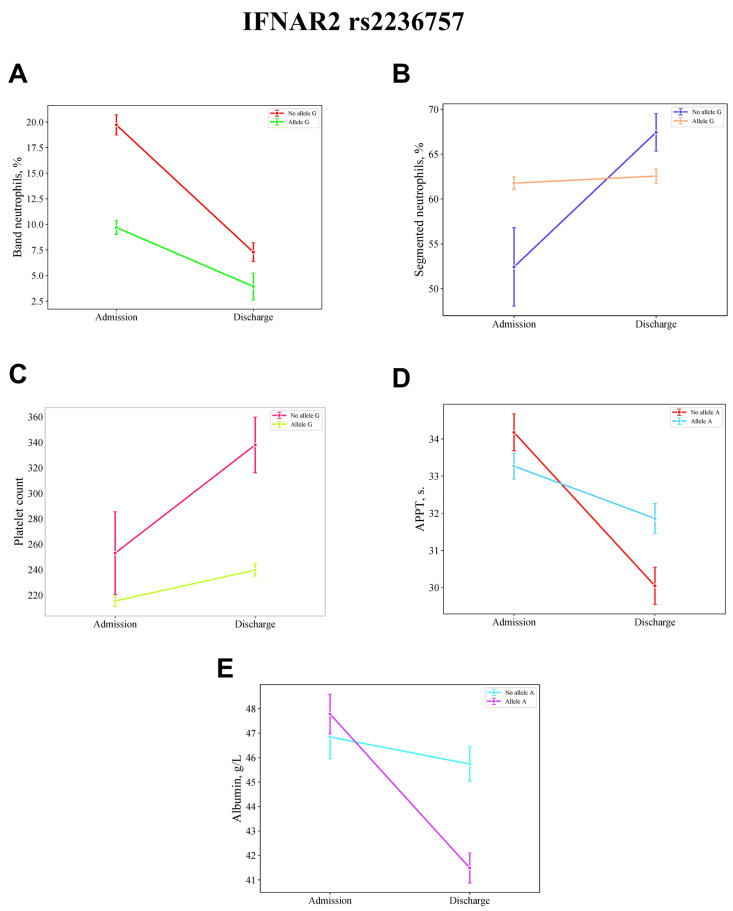
Repeated measures ANOVA demonstrates the impact of the presence of the IFNAR2 rs2236757 G and A alleles on the temporal dynamics of band neutrophil (**A**), segmented neutrophil (**B**), platelet count (**C**), APTT (**D**), and albumin (**E**) levels. Data are presented as mean ± standard error (SE).

**Figure 2:**
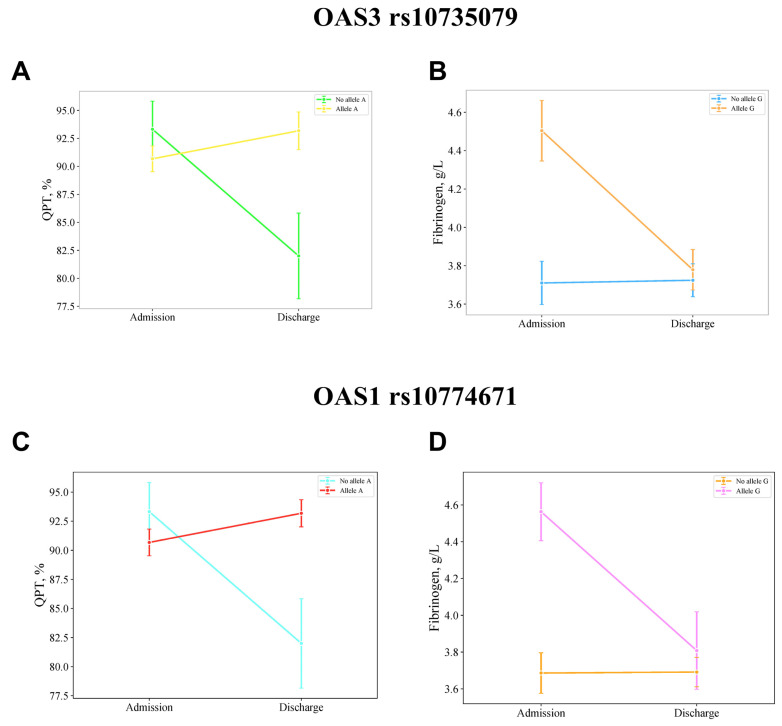
Repeated measures ANOVA demonstrates the impact of the OAS3 rs10735079 and OAS1 rs10774671 A allele on the temporal changes in quick plasma clotting time (QPT) levels (**A**,**C**) and the OAS3 rs10735079 and OAS1 rs10774671 G allele on fibrinogen levels (**B**,**D**). Data are presented as mean ± standard error (SE).

Figure 1 and Figure 2 provide a detailed visualization of the dynamic changes in laboratory parameters over the course of hospitalization, stratified by the presence of specific genetic polymorphisms.

Mean values and standard deviations for these parameters are presented in Table 4, while pairwise comparisons of mean differences are detailed in Table 5.

Patients lacking the *IFNAR2 rs2236757* G allele at admission exhibited higher band neutrophil (MD = 10.022, 95% CI: 2.402, 17.642) and fibrinogen levels (MD = 1.908, 95% CI: 0.362, 3.454) compared with those with the G allele. These patients continued to show higher band neutrophil (MD = 12.429, 95% CI: 4.766, 20.091) and fibrinogen levels (MD = 1.643, 95% CI: 0.092, 3.194) and lower platelet count (MD = −85.000, 95% CI: −149.063, −20.937) compared with those without the G allele at discharge. Additionally, these patients exhibited higher band neutrophil (MD = 15.776, 95% CI: 8.156, 23.396) and fibrinogen levels (MD = 2.193, 95% CI: 0.647, 3.739) compared with those with the G allele at discharge.

The presence of the IFNAR2 rs2236757 G allele at admission was associated with lower platelet count (MD = −122.569, 95% CI: −215.494, −29.645) compared with those without the G allele at discharge, higher band neutrophil levels (MD = 5.754, 95% CI: 3.239, 8.268) and lower platelet count (MD = −24.292, 95% CI: −45.316, −3.269) compared with those with the G allele at discharge, and higher platelet count (MD = 98.277, 95% CI: 5.352, 191.201) compared with those without the G allele at discharge.

Patients lacking the *IFNAR2 rs2236757* A allele at admission exhibited a higher activated partial thromboplastin time (APTT) level (MD = 4.129, 95% CI: 1.897, 6.361) compared with those without the A allele at discharge. Conversely, the presence of the A allele at admission was associated with a higher albumin level (MD = 6.293, 95% CI: 2.592, 9.993) compared with those with the A allele at discharge.

Patients carrying the G allele in both *OAS3 rs10735079* and *OAS1 rs10774671* polymorphisms at admission showed higher fibrinogen levels compared with those with the A allele at discharge (MD = 0.725, 95% CI: 0.088, 1.362 for *OAS3* and MD = 0.755, 95% CI: 0.103, 1.406 for *OAS1*).

### 3.3. Genetic Determinants of Laboratory Parameter Differences

A comparative analysis of clinical and laboratory outcomes at hospital discharge in patients treated with Paxlovid is presented in Table 6.

**Figure 3:**
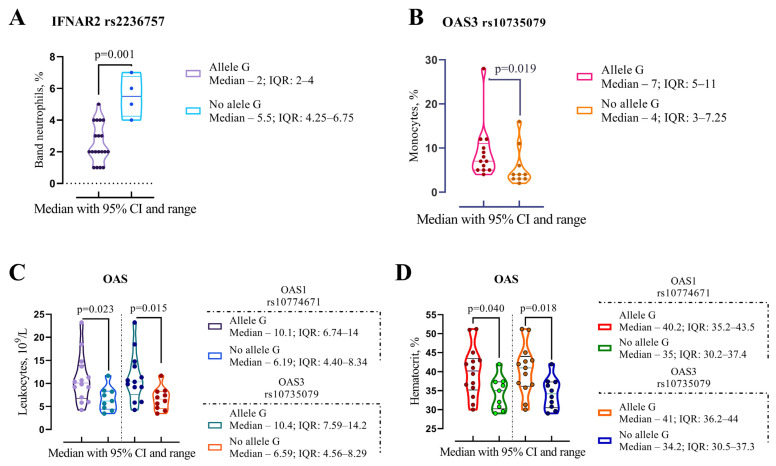
Comparison of median clinical and laboratory findings at hospital discharge in COVID-19 patients treated with Paxlovid, stratified by the presence of the G allele. Panel (**A**): IF-NAR2 rs2236757—band neutrophil levels. Panel (**B**): OAS3 rs10735079—monocyte levels. Panels (**C**,**D**): OAS3 rs10735079 and OAS1 rs10774671—leukocyte and hematocrit levels. Median values with interquartile ranges (IQRs) are reported. Statistical significance was determined using the Wilcoxon matched pairs test.

Patients carrying the *IFNAR2 rs2236757* G allele exhibited significantly lower band neutrophil counts (2%, IQR 2–4 vs. 5.5%, IQR 4.25–6.75, *p* = 0.001) compared with those without the G allele (Figure 3A).

Patients with the *OAS3 rs10735079* G allele demonstrated elevated levels of leukocytes (10.4 × 10^9^/L, IQR 7.59–14.2 vs. 6.59 × 10^9^/L, IQR 4.56–8.29, *p* = 0.015), monocytes (7%, IQR 5–11 vs. 4%, IQR 3–7.25, *p* = 0.019), and hematocrit (41%, IQR 36.2–44 vs. 34.2%, IQR 30.5–37.3, *p* = 0.018) compared with those without the G allele (Figure 1B–D). Additionally, patients with the *OAS1 rs10774671* G allele exhibited higher leukocyte (10.1 × 10^9^/L, IQR 6.74–14 vs. 6.19 × 10^9^/L, IQR 4.40–8.34, *p* = 0.023) and hematocrit (40.2%, IQR 35.2–43.5 vs. 35%, IQR 30.2–37.4, *p* = 0.040) levels compared with those without the G allele (Figure 3C,D).

**Figure 4:**
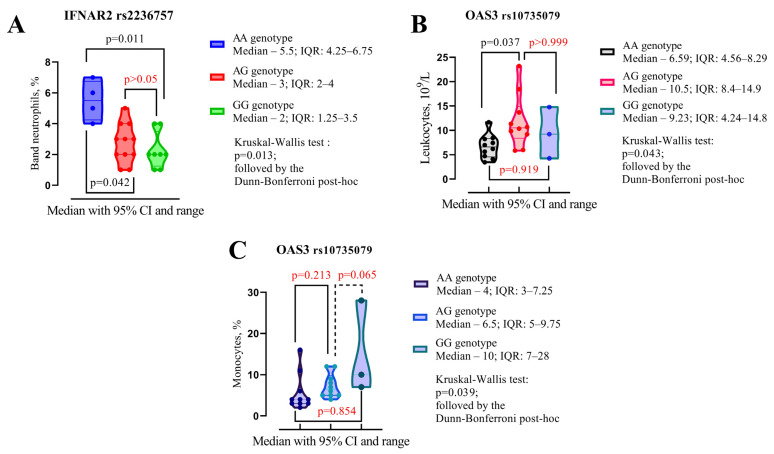
A Kruskal–Wallis test with Dunn’s multiple comparisons post hoc analysis was used to compare the median laboratory findings at discharge among patients with different genotypes of IFNAR2 rs2236757, OAS1 rs10774671, and OAS3 rs10735079. (**A**) shows the segmented neutrophil levels across the AA, AG, and GG genotypes of the IFNAR2 rs2236757 polymorphism. (**B**) shows the leukocyte levels across the AA, AG, and GG genotypes of the OAS3 rs10735079 polymorphism. (**C**) shows the monocyte levels across the AA, AG, and GG genotypes of the OAS1 rs10774671 polymorphism.

We further compared patients with different SNP genotypes (Figure 4). Patients with the IFNAR2 rs2236757 AA genotype exhibited higher band neutrophil counts compared with those with the AG genotype (5.5% IQR 4.25–6.75 vs. 3%, 2–4, *p* = 0.042) and the GG genotype (5.5% IQR 4.25–6.75 vs. 2%, 1.25–3.5, *p* = 0.011) (Figure 4A).

Patients with the *OAS1 rs10774671* AA genotype demonstrated lower leukocyte levels compared with those with the AG genotype (6.59 × 10^9^/L, IQR 4.56–8.29 vs. 10.59 × 10^9^/L, IQR 8.4–14.9, *p* = 0.037) (Figure 2B). Patients with the *OAS3 rs10735079* genotype exhibited significant differences in monocyte levels between genotypes as assessed by the Kruskal–Wallis test (*p* = 0.039). However, no significant differences were observed when analyzed using Dunn’s multiple comparisons test (Figure 4C).

### 3.4. Alleles, Genotypes, and Clinical Outcomes

To investigate the potential impact of specific alleles on laboratory outcome variability during hospitalization in COVID-19 patients receiving Paxlovid treatment, a comparative analysis was conducted (Table 7).

At discharge, patients with the *IFNAR2 rs2236757* G allele exhibited higher SpO2 (98%, IQR 97–98 vs. 96%, IQR 92–97, *p* = 0.019), segmented neutrophil counts (66%, IQR 52–78 vs. 55%, IQR 46–75, *p* = 0.029), and AST (30.8 mmol/L, IQR 23.3–94.1 vs. 22.6 mmol/L, IQR 16.6–25.8, *p* = 0.014) levels. Conversely, they displayed lower eosinophil counts (1%, IQR 0–1 vs. 1%, IQR 1–2, *p* = 0.048), hematocrit (37%, IQR 32–42.7 vs. 40%, IQR 34.2–45, *p* = 0.040), APTT (29.8 s, IQR 25–33.7 vs. 33.2 s, IQR 29.4–37, *p* = 0.025), and total bilirubin (11.2 mmol/L, IQR 10.5–13.5 vs. 13.7 mmol/L, IQR 10.8–19.1, *p* = 0.029) levels. Patients with the *IFNAR2 rs2236757* A allele demonstrated higher platelet counts (215 × 10^9^/L, IQR 166–244 vs. 173 × 10^9^/L, IQR 142–204, *p* = 0.041) and lower ESR (5 mm/h, IQR 4–6 vs. 7 mm/h, IQR 4–11, *p* = 0.021), creatinine (92 mmol/L, IQR 84–109 vs. 103 mmol/L, IQR 95–117, *p* = 0.044), and albumin (43 g/L, IQR 37–51 vs. 50 g/L, IQR 45–57, *p* = 0.023) levels.

Patients with the *ACE2 rs2074192* C allele exhibited higher SpO_2_ (98%, IQR 97–98 vs. 96%, IQR 93.5–98, *p* = 0.019), segmented neutrophil counts (66%, IQR 51.3–75.8 vs. 57%, IQR 46–71.3, *p* = 0.016), and AST (32.3 mmol/L, IQR 24.2–81.2 vs. 22.4 mmol/L, IQR 17.2–26.3, *p* = 0.004) levels along with lower APTT (29.8 s, IQR 25.5–34.9 vs. 33.3 s, IQR 29.7–37.1, *p* = 0.027) and fibrinogen (3.33 g/L, IQR 2.76–3.99 vs. 3.99 g/L, IQR 3.55–4.94, *p* = 0.017) levels. In contrast, patients with the *ACE2 rs2074192* T allele displayed lower total bilirubin (10.8 mmol/L, IQR 10.2–12 vs. 12.9 mmol/L, IQR 10.7–17.8, *p* = 0.028) and ALP (136 mmol/L, IQR 94.3–148 vs. 148 mmol/L, IQR 125–165, *p* = 0.025) levels.

Patients with the *OAS3 rs10735079* A allele exhibited higher segmented neutrophil counts (70.5%, IQR 53.3–77.3 vs. 61%, IQR 47.5–73.8, *p* = 0.027) and AST (30.1 mmol/L, IQR 24.5–67.4 vs. 22.7 mmol/L, IQR 19.4–27.3, *p* = 0.006) levels but lower hematocrit (37.2%, IQR 31.5–42 vs. 38.6%, IQR 34.3–43.5, *p* = 0.021), APTT (29.8 s, IQR 26.9–35.2 vs. 34.2 s, IQR 30–37.2, *p* = 0.033), and ALP (113 mmol/L, IQR 93.8–144 vs. 136 mmol/L, IQR 115–152, *p* = 0.025) levels. Conversely, patients with the *OAS3 rs10735079* G allele displayed lower eosinophil count (1%, IQR 0–1 vs. 1%, IQR 1–2.5, *p* = 0.046), fibrinogen (3.33 g/L, IQR 2.11–3.88 vs. 3.99 g/L, IQR 3.55–4.1, *p* = 0.031), and total bilirubin (10.7 mmol/L, IQR 10.3–13.2 vs. 12.9 mmol/L, IQR 11.1–20.6, *p* = 0.021) levels.

Patients with the *OAS1 rs10774671* A allele exhibited higher segmented neutrophil counts (70.5%, IQR 53.3–77.3 vs. 47.5%, IQR 61–73.8, *p* = 0.027) and AST (30.1 mmol/L, IQR 24.5–67.4 vs. 22.7 mmol/L, IQR 19.4–27.3, *p* = 0.006) levels and lower hematocrit (37.2%, IQR 31.5–42 vs. 38.6%, IQR 34.3–43.5, *p* = 0.021) and ALP (113 mmol/L, IQR 93.8–144 vs. 136 mmol/L, IQR 115–152, *p* = 0.025) levels. Patients with the *OAS1 rs10774671* G allele showed lower eosinophil count (1%, IQR 0–1 vs. 1%, IQR 1–2.25, *p* = 0.035) and fibrinogen (3.33 g/L, IQR 2.27–3.99 vs. 3.99 g/L, IQR 3.55–4.26, *p* = 0.046) and total bilirubin (10.9 mmol/L, IQR 10.4–13.7 vs. 13.3 mmol/L, IQR 11.2–22, *p* = 0.014) levels.

## 4. Discussion

*IFNAR2*, a transmembrane receptor, is a component of the type I interferon (*IFN*) receptor complex, recognizing *IFN-α* and *IFN-β* [85,92]. The binding of *IFN-I* to *IFNAR* initiates a signaling cascade, leading to the expression of interferon-stimulated genes (ISGs) with antiviral, antiproliferative, and immunomodulatory functions [93]. One critical ISG is the RNA-activated protein kinase (PKR). Additionally, *IFN* activates the oligoadenylate synthetase (*OAS*) family proteins (*OAS1*, 2, and 3), which catalyze the synthesis of 2′-5′ oligoadenylate (2′-5′A). Subsequently, 2′-5′A activates RNase L, resulting in viral RNA degradation [93]. Genetic variations within *IFNAR2*, *OAS1*, and *OAS3* could potentially disrupt this signaling pathway, leading to decreased protein abundance, impaired receptor internalization, or altered ligand interactions, thereby exacerbating the severity of COVID-19 [65,77,83].

All four SNPs (*IFNAR2*, *OAS1*, *OAS2*, and *ACE2*) investigated are non-synonymous SNPs (nsSNPs), resulting in alterations to the amino acid sequence of the encoded protein. The variants rs2074192, rs2236757, and rs10735079 are located within introns, whereas rs10774671 is a splice acceptor variant. Although our study focused on a Ukrainian population, extrapolating these findings to other ethnicities necessitates further investigation. To this end, we compared our data with publicly available allele frequencies for the European population from Ensembl.org (https://www.ensembl.org/). No significant discrepancies in allele distribution were identified between the two populations.

Nirmatrelvir, an orally administered protease inhibitor, binds to the catalytic dyad of Mpro via its nitrile moiety [94,95,96]. Ritonavir, a tripeptide, inhibits HIV protease by binding to its active site [95,97]. Nirmatrelvir demonstrates potent inhibitory activity against Mpro in all seven human coronavirus genotypes, including alpha-coronaviruses (HCoV-NL63 and HCoV-229E) and beta-coronaviruses (MERS-CoV, SARS-CoV-1, SARS-CoV-2, HCoV-OC43, and HCoV-HKU1) [97,98].

The findings from this study provide valuable insights into the role of genetic polymorphisms in shaping the clinical outcomes of COVID-19 patients treated with Paxlovid. These results align with, and in some cases extend, findings from previous studies that have explored the impact of host genetics on disease progression and treatment response in COVID-19 [65,99,100].

The association between the *IFNAR2 rs2236757* G allele and altered inflammatory and coagulation markers observed in our study is consistent with previous research indicating that polymorphisms in the interferon receptor pathway play a significant role in modulating immune responses [101,102]. Similarly, our findings suggest that the G allele may contribute to an elevated inflammatory state, which could exacerbate disease severity [103,104]. However, while our study identified significant changes in hematological parameters, such as neutrophils and fibrinogen, further studies are needed to explore whether these variations directly affect the clinical outcomes of Paxlovid treatment.

The role of *OAS1* and *OAS3* polymorphisms in influencing immune and coagulation responses, as observed in this study, is also supported by prior research on type I interferons [105,106,107]. Polymorphisms in these genes have been shown to impact the production of interferons and subsequent antiviral immunity, which is crucial in the early stages of infection [108,109,110]. For example, researches demonstrated that *OAS1* and *OAS3* polymorphisms influence the innate immune response and the body’s ability to mount an effective defense against SARS-CoV-2 [111,112,113]. Our study builds on this by suggesting that these polymorphisms also affect coagulation pathways, potentially influencing the severity of complications in COVID-19 patients.

In accordance with Hardy–Weinberg equilibrium principles, the observed allele frequencies in our sample were consistent with those reported for the general population. While the statistical power of the current sample size was sufficient to discern associations between the investigated alleles and the clinical and laboratory parameters, future research endeavors should consider increasing the sample size to adequately address the potential influence of additional covariates, such as age, sex, and comorbidity, as well as their potential interactions.

The relationship between immune markers and the genetic variants we studied, particularly the influence of *OAS1* and *OAS3* alleles on leukocyte, monocyte, and hematocrit levels, is also in line with previous findings. For example, studies highlighted the crucial role of inflammatory biomarkers such as leukocyte counts and neutrophil-to-lymphocyte ratio in predicting COVID-19 severity [114,115,116]. The elevation of these markers in individuals with specific *OAS3* and *OAS1* alleles in our study suggests a potential link between genetic susceptibility and heightened immune activation, which may influence clinical outcomes and treatment efficacy [117,118,119]. *ACE2* remains a critical receptor for SARS-CoV-2 entry; its genetic variation may not play as central a role in influencing the clinical effects of antiviral treatments like Paxlovid, at least in the context of the immune and coagulation parameters studied here [120,121,122,123].

**Figure 5:**
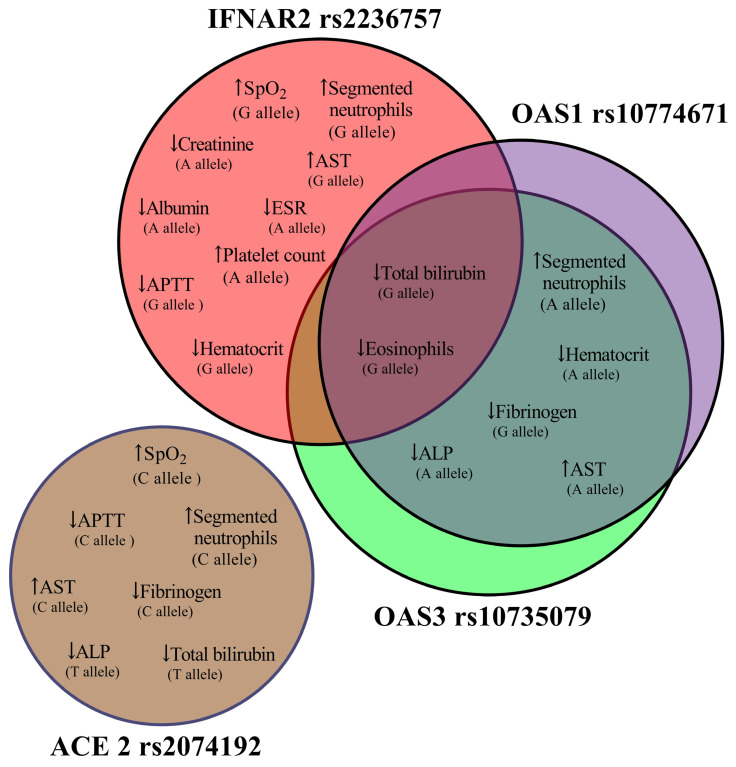
A Venn diagram illustrates changes in blood parameters between admission and dis-charge, categorized by SPPs’ (IFNAR2 rs2236757, OAS3 rs10735079, OAS1 rs1077467, and ACE2 rs2074192) presence of different alleles.

When comparing patients at hospitalization and discharge, we identified statistically significant differences in certain outcomes based on the studied polymorphisms (Figure 5).

Regarding oxygen saturation, we observed increases in patients with the *IFNAR2 rs2236757* G allele and *ACE2 rs2074192* C allele. This suggests a potential link between this variant and a more robust initial antiviral response, possibly leading to better oxygenation. This observation aligns with the broader understanding of type I interferons’ crucial role in controlling viral replication and modulating the immune response in viral infections, including SARS-CoV-2 (125,126).

Additionally, patients with the *IFNAR2 rs2236757* G allele and *OAS3 rs10735079* and *OAS1 rs1077467* A alleles exhibited elevated segmented neutrophil counts and AST levels and decreased hematocrit. This combination of findings could indicate a more pronounced inflammatory response in these individuals. This constellation of findings could reflect a heightened inflammatory state, potentially driven by an overactive innate immune response. Elevated AST, a marker of liver injury, may indicate systemic inflammation or direct viral effects on the liver. The observed decrease in hematocrit, potentially indicative of anemia, could be a consequence of chronic inflammation or other disease-related factors.

The G allele of the three interferon-related SNPs was associated with lower eosinophil and total bilirubin levels. The reduction in eosinophils might reflect a suppression of Th2-mediated immune responses, potentially influenced by the interferon signaling pathways. Lower bilirubin levels, while potentially multifactorial, could be related to altered liver function or reduced hemolysis.

Fibrinogen and ALP levels were decreased in patients with the *OAS3 rs10735079* and *OAS1 rs1077467* G and A alleles, respectively, and with the *ACE2 rs2074192* C and T alleles. Other changes linked to the *IFNAR2 rs2236757* A allele included increased platelet count and decreased creatinine, albumin, and ESR levels, while the G allele demonstrated a decreased APTT level. Patients with the *ACE2 rs2074192* C allele exhibited elevated segmented neutrophils and AST levels and decreased APTT, and the T allele was associated with lower total bilirubin levels. These findings are in agreement with previous research, which has highlighted the influence of genetic polymorphisms in interferon-related genes (e.g., *IFNAR2*, *OAS3*, *OAS1*) on immune response modulation and disease outcomes in COVID-19 [124,125,126,127,128].

In our prior investigation, we presented the findings of a study assessing the efficacy of Paxlovid treatment in patients diagnosed with COVID-19 [17]. Notably, the administration of this antiviral agent was associated with a statistically significant reduction in the median duration of hospital stay (9 days vs. 11 days, *p* < 0.05). Furthermore, a positive correlation was observed between Paxlovid treatment and SpO_2_. The presence of comorbid conditions, including MAFLD, obesity, and chronic obstructive pulmonary disease (COPD), was documented in the patient cohort. Statistical analysis indicated that patient age and gender did not exert a significant influence on the length of hospitalization, whereas T2DM, the severity of the COVID-19 infection, and the presence of pneumonia were identified as factors significantly impacting the duration of hospital stay.

In the present investigation, the primary objective was to elucidate the impact of specific genetic polymorphisms on the dynamic profile of clinical and laboratory parameters in individuals diagnosed with COVID-19, with a particular emphasis on those receiving Paxlovid. Nevertheless, subsequent research endeavors should prioritize the expansion of the study cohort to concurrently evaluate the interplay between the presence of defined alleles and therapeutic intervention as determinants influencing the dynamics of laboratory parameters.

## 5. Limitations

The present study is subject to several limitations. The sample size of 72 participants may limit the statistical power of the study, especially for subgroup analyses and the detection of smaller effect sizes. Nevertheless, the Power Analysis we conducted demonstrated that the sample size is sufficient for reliable statistical conclusions. The study population was predominantly of Ukrainian ethnicity, which may limit the generalizability of the findings to other populations with different genetic backgrounds. This study was conducted at a single center, which may introduce potential biases related to patient selection and treatment protocols. The study did not include long-term follow-up of patients, which could provide valuable information on the long-term consequences of COVID-19 and the potential impact of genetic factors on post-acute COVID-19 syndrome. The study did not account for all potential confounding factors, such as socioeconomic status, comorbidities, and lifestyle factors, which could influence disease progression and laboratory parameters. The scope of this study was circumscribed to a specific set of four SNPs. These SNPs were selected based on prior evidence from the scientific literature demonstrating their association with increased severity of COVID-19 infection.

## 6. Conclusions

This study highlights the significant impact of genetic variations in *IFNAR2*, *OAS1*, *OAS3*, and *ACE2* on the clinical and laboratory outcomes of COVID-19 patients receiving Paxlovid treatment. The findings suggest that these SNPs may influence the immune response, liver function, and coagulation parameters.

Polymorphisms in the interferon-related genes *IFNAR2*, *OAS1*, and *OAS3* were associated with variations in inflammatory and coagulation markers. Notably, the *IFNAR2 rs2236757* G allele was linked to alterations in band neutrophil, platelet, and fibrinogen levels. *OAS1* and *OAS3* polymorphisms influenced coagulation parameters such as QPT and fibrinogen. These findings highlight the potential role of these genes in modulating the immune response and coagulation pathways during COVID-19 infection.

Further research is necessary to elucidate the precise mechanisms underlying these associations and to explore the potential therapeutic implications of targeting these genetic markers for personalized treatment strategies in COVID-19 patients.

## Figures and Tables

**Table 1:** Initial Characteristics.

	Paxlovid Treatment (*n* = 23)	Standard Treatment (*n* = 49)	*p*-Value ^a^
Age, median (IQR) ^b^	64 (46–71)	66 (51–72)	0.721
Male, No. (%)	12 (52.17%)	31 (63.26%)	0.339
BMI, kg/m^2^	26.5 (2.09–29.7)	26.1 (23.84–30.97)	0.717
Duration of hospital stay, days	9 (7–11)	11 (9–14)	0.001
COVID-19 severity (moderate/severe/critical), *n*	18/4/1	24/21/4	0.062
Need for oxygen supply, *n* (%)	5 (1.17%)	15 (30.61%)	0.575

^a^ Fisher exact, chi-square, or Mann–Whitney U test, as appropriate; ^b^ data are presented as medians (interquartile range). Abbreviation: IQR—interquartile range.

**Table 2:** Assessment of Hardy–Weinberg equilibrium.

Paxlovid Treatment									
Genotype	ACE2 rs2074192		Genotype	IFNAR2 rs2236757		OAS3 rs10735079		OAS1 rs10774671	
	Expected	Observed		Expected	Observed	Expected	Observed	Expected	Observed
CC	14.88	15	AA	3.92	4	9.141	9	9.78	10
CT	7.24	7	AG	11.15	11	10.72	11	10.43	10
TT	0.88	1	GG	7.92	8	3.14	3	2.78	3
	χ^2^ = 0.025; *p* = 0.874			χ^2^ = 0.004; *p* = 0.949		χ^2^ = 0.039; *p* = 0.841		χ^2^ = 0.016; *p* = 0.899	
Standard Treatment									
CC	26.45	25	AA	4.29	3	19.61	20	21.55	23
CT	19.10	22	AG	20.42	23	22.78	22	21.59	19
TT	3.45	2	GG	24.29	23	6.61	7	5.56	7
	χ^2^ = 1.128; *p* = 0.569			χ^2^ = 0.783; *p* = 0.676		χ^2^ = 0.057; *p* = 0.972		χ^2^ = 0.853; *p* = 0.653	

Deviations from Hardy–Weinberg equilibrium (HWE) can signal inbreeding, population stratification, genotyping errors, or genetic associations in affected individuals. HWE is typically assessed using a chi-square goodness-of-fit test.

**Table 3:** Repeated measures ANOVA with between- and within-subject effects.

IFNAR2 rs2236757							
Repeated Measures	Within-Subject Effect				Between-Subject Effect		
	Interaction	F	*p*-Value	η^2^_p_	F	*p*-Value	η^2^_p_
Band neutrophils, %	Allele G × Time	5.051	*p* = 0.028	0.067	7.632	*p* = 0.007	0.098
Segmented neutrophils, %		5.688	*p* = 0.020	0.075	0.264	*p* = 0.609	0.004
Platelet count		5.977	*p* = 0.017	0.079	4.468	*p* = 0.038	0.060
Fibrinogen, g/L		5.101	*p* = 0.027	0.068	6.263	*p* = 0.015	0.082
APPT, s.	Allele A × Time	6.236	*p* = 0.015	0.082	0.172	*p* = 0.679	0.002
Albumin, g/L		6.258	*p* = 0.015	0.082	0.783	*p* = 0.379	0.011
OAS3 rs10735079							
QPT, %	Allele A × Time	5.380	*p* = 0.023	0.071	0.570	*p* = 0.453	0.008
Fibrinogen, g/L	Allele G × Time	4.128	*p* = 0.046	0.056	1.951	*p* = 0.167	0.027
OAS1 rs10774671							
QPT, %	Allele A × Time	5.380	*p* = 0.023	0.071	0.570	*p* = 0.453	0.008
Fibrinogen, g/L	Allele G × Time	4.452	*p* = 0.038	0.060	2.748	*p* = 0.102	0.038

Inter-group differences can be modeled as a between-subjects factor, while intra-group variations resulting from repeated measures can be modeled as a within-subjects factor.

**Table 4:** Descriptive statistics for interaction effects.

Band Neutrophils, %	*IFNAR2 rs2236757 Allele G*	Mean	SD	SE
Admission	*No allele G*	19.714	19.576	7.399
	*Allele G*	9.692	6.685	0.829
Discharge	*No allele G*	7.286	6.626	2.504
	*Allele G*	3.938	5.166	0.641
Segmented Neutrophils, %				
Admission	*No allele G*	52.429	22.150	8.372
	*Allele G*	61.769	12.427	1.541
Discharge	*No allele G*	67.429	11.297	4.270
	*Allele G*	62.554	13.104	1.625
Platelet Count				
Admission	*No allele G*	253.000	173.305	65.503
	*Allele G*	215.431	66.937	8.302
Discharge	*No allele G*	338.000	169.051	63.895
	*Allele G*	239.723	80.180	9.945
Fibrinogen, g/L				
Admission	*No allele G*	5.896	2.809	1.062
	*Allele G*	3.988	1.511	0.187
Discharge	*No allele G*	4.253	1.621	0.613
	*Allele G*	3.703	1.147	0.142
APPT, s.	*IFNAR2 rs2236757 Allele A*	Mean	SD	SE
Admission	*No allele A*	34.177	5.459	0.980
	*Allele A*	33.268	4.522	0.706
Discharge	*No allele A*	30.048	5.457	0.980
	*Allele A*	31.860	5.116	0.799
Albumin, g/L				
Admission	*No allele A*	46.839	9.256	1.662
	*Allele A*	47.780	10.398	1.624
Discharge	*No allele A*	45.742	8.000	1.437
	*Allele A*	41.488	7.916	1.236
QPT, %	*OAS3 rs10735079 Allele A*	Mean	SD	SE
Admission	*No allele A*	93.320	16.194	5.121
	*Allele A*	90.676	18.490	2.348
Discharge	*No allele A*	82.000	24.119	7.627
	*Allele A*	93.182	18.453	2.344
Fibrinogen, g/L				
Admission	*No allele G*	3.710	1.237	0.226
	*Allele G*	4.504	1.982	0.306
Discharge	*No allele G*	3.724	0.943	0.172
	*Allele G*	3.779	1.361	0.210
QPT, %	*OAS1 rs10774671 Allele A*	Mean	SD	SE
Admission	*No allele A*	93.320	16.194	5.121
	*Allele A*	90.676	18.490	2.348
Discharge	*No allele A*	82.000	24.119	7.627
	*Allele A*	93.182	18.453	2.344
Fibrinogen, g/L				
Admission	*No allele G*	3.686	1.247	0.220
	*Allele G*	4.563	1.991	0.315
Discharge	*No allele G*	3.691	0.905	0.160
	*Allele G*	3.808	1.398	0.221

Data are presented as mean ± standard deviation (SD) and standard error (SE) of the mean.

**Table 5:** Post hoc tests with Bonferroni correction.

Band Neutrophils, %							
			95% CI for Mean Difference				
**IFNAR2 rs2236757 Allele G** **×** **Time**		**Mean Difference**	**Lower**	**Upper**	**SE**	**t**	***p*** **_bonf._**
No allele G, Admission	Allele G, Admission	10.022	2.402	17.642	2.839	3.530	0.004
	No allele G, Discharge	12.429	4.766	20.091	2.822	4.404	<0.001
	Allele G, Discharge	15.776	8.156	23.396	2.839	5.557	<0.001
Allele G, Admission	No allele G, Discharge	2.407	−5.214	10.027	2.839	0.848	1.000
	Allele G, Discharge	5.754	3.239	8.268	0.926	6.213	<0.001
No allele G, Discharge	Allele, G, Discharge	3.347	−4.273	10.967	2.839	1.179	1.000
**Segmented Neutrophils, %**							
No allele G, Admission	Allele G, Admission	−9.341	−23.474	4.793	5.271	−1.772	0.473
	No allele G, Discharge	−15.000	−30.378	0.378	5.664	−2.649	0.060
	Allele G, Discharge	−10.125	−24.259	4.008	5.271	−1.921	0.342
Allele G, Admission	No allele G, Discharge	−5.659	−19.793	8.474	5.271	−1.074	1.000
	Allele G, Discharge	−0.785	−5.831	4.262	1.859	−0.422	1.000
No allele G, Discharge	Allele, G, Discharge	4.875	−9.259	19.008	5.271	0.925	1.000
**Platelet Count**							
No allele G, Admission	Allele G, Admission	37.569	−55.355	130.494	34.448	1.091	1.000
	No allele G, Discharge	−85.000	−149.063	−20.937	23.593	−3.603	0.004
	Allele G, Discharge	13.277	−79.648	106.201	34.448	0.385	1.000
Allele G, Admission	No allele G, Discharge	−122.569	−215.494	−29.645	34.448	−3.558	0.004
	Allele G, Discharge	−24.292	−45.316	−3.269	7.743	−3.138	0.015
No allele G, Discharge	Allele, G, Discharge	98.277	5.352	191.201	34.448	2.853	0.032
**Fibrinogen, g/L**							
No allele G, Admission	Allele G, Admission	1.908	0.362	3.454	0.576	3.314	0.007
	No allele G, Discharge	1.643	0.092	3.194	0.571	2.876	0.032
	Allele G, Discharge	2.193	0.647	3.739	0.576	3.809	0.001
Allele G, Admission	No allele G, Discharge	−0.265	−1.811	1.280	0.576	−0.460	1.000
	Allele G, Discharge	0.285	−0.224	0.794	0.187	1.521	0.796
No allele G, Discharge	Allele, G, Discharge	0.550	−0.995	2.096	0.576	0.956	1.000
**APPT, s**							
			95% CI for Mean Difference				
**IFNAR2 rs2236757 Allele A × Time**		**Mean Difference**	**Lower**	**Upper**	**SE**	**t**	***p*** **_bonf._**
No allele A, Admission	Allele A, Admission	0.909	−2.361	4.179	1.216	0.748	1.000
	No allele A, Discharge	4.129	1.897	6.361	0.822	5.022	<0.001
	Allele A, Discharge	2.317	−0.953	5.588	1.216	1.906	0.356
Allele A, Admission	No allele A, Discharge	3.220	−0.050	6.490	1.216	2.649	0.056
	Allele A, Discharge	1.408	−0.533	3.349	0.715	1.970	0.317
No allele A, Discharge	Allele, A, Discharge	−1.812	−5.082	1.459	1.216	−1.490	0.835
**Albumin, g/L**							
No allele A, Admission	Allele A, Admission	−0.942	−6.693	4.809	2.140	−0.440	1.000
	No allele A, Discharge	1.097	−3.159	5.353	1.567	0.700	1.000
	Allele A, Discharge	5.351	−0.400	11.102	2.140	2.500	0.083
Allele A, Admission	No allele A, Discharge	2.039	−3.713	7.790	2.140	0.952	1.000
	Allele A, Discharge	6.293	2.592	9.993	1.363	4.617	<0.001
No allele A, Discharge	Allele, A, Discharge	4.254	−1.497	10.005	2.140	1.988	0.296
**QPT, %**							
**OAS3 rs10735079 Allele A × Time**		**Mean Difference**	**Lower**	**Upper**	**SE**	**t**	***p*** **_bonf._**
No allele A, Admission	Allele A, Admission	2.644	−14.536	19.824	6.390	0.414	1.000
	No allele A, Discharge	11.320	−3.699	26.339	5.531	2.046	0.267
	Allele A, Discharge	0.138	−17.042	17.318	6.390	0.022	1.000
Allele A, Admission	No allele A, Discharge	8.676	−8.504	25.856	6.390	1.358	1.000
	Allele A, Discharge	−2.506	−8.538	3.526	2.221	−1.128	1.000
No allele A, Discharge	Allele, A, Discharge	−11.182	−28.362	5.998	6.390	−1.750	0.498
**Fibrinogen, g/L**							
**OAS3 rs10735079 Allele G × Time**		**Mean Difference**	**Lower**	**Upper**	**SE**	**t**	***p*** **_bonf._**
No allele G, Admission	Allele G, Admission	−0.794	−1.744	0.157	0.354	−2.242	0.161
	No allele G, Discharge	−0.014	−0.768	0.740	0.278	−0.050	1.000
	Allele G, Discharge	−0.069	−1.019	0.882	0.354	−0.194	1.000
Allele G, Admission	No allele G, Discharge	0.780	−0.171	1.731	0.354	2.203	0.178
	Allele G, Discharge	0.725	0.088	1.362	0.235	3.089	0.017
No allele G, Discharge	Allele, G, Discharge	−0.055	−1.006	0.896	0.354	−0.155	1.000
**QPT, %**							
**OAS1 rs10774671 Allele A × Time**		**Mean Difference**	**Lower**	**Upper**	**SE**	**t**	***p*** **_bonf._**
No allele A, Admission	Allele A, Admission	2.644	−14.536	19.824	6.390	0.414	1.000
	No allele A, Discharge	11.320	−3.699	26.339	5.531	2.046	0.267
	Allele A, Discharge	0.138	−17.042	17.318	6.390	0.022	1.000
Allele A, Admission	No allele A, Discharge	8.676	−8.504	25.856	6.390	1.358	1.000
	Allele A, Discharge	−2.506	−8.538	3.526	2.221	−1.128	1.000
No allele A, Discharge	Allele, A, Discharge	−11.182	−28.362	5.998	6.390	−1.750	0.498
**Fibrinogen, g/L**							
**OAS1 rs10774671 Allele G × Time**		**Mean Difference**	**Lower**	**Upper**	**SE**	**t**	***p*** **_bonf._**
No allele G, Admission	Allele G, Admission	−0.877	−1.816	0.062	0.350	−2.507	0.081
	No allele G, Discharge	−0.005	−0.733	0.724	0.268	−0.018	1.000
	Allele G, Discharge	−0.122	−1.061	0.817	0.350	−0.349	1.000
Allele G, Admission	No allele G, Discharge	0.872	−0.067	1.811	0.350	2.494	0.084
	Allele G, Discharge	0.755	0.103	1.406	0.240	3.145	0.015
No allele G, Discharge	Allele, G, Discharge	−0.117	−1.056	0.822	0.350	−0.335	1.000

**Table 6:** Impact of SNP alleles on clinical and laboratory outcomes at discharge in Paxlovid-treated COVID-19 patients.

	IFNAR2 rs2236757		
	No Allele G (n = 4)	Allele G (n = 19)	*p*-Value ^a^
Band neutrophils, % (IQR)	5.5 (4.25–6.75)	2 (2–4)	*p* = 0.001
	**OAS3 rs10735079**		
	No Allele G (n = 10)	Allele G (n = 13)	*p*-Value
Leukocytes, 10^9^/L	6.59 (4.56–8.29)	10.4 (7.59–14.2)	*p* = 0.015
Monocytes, %	4 (3–7.25)	7 (5–11)	*p* = 0.019
Hematocrit, %	34.2 (30.5–37.3)	41 (36.2–44)	*p* = 0.018
	**OAS1 rs10774671**		
	No Allele G (n = 9)	Allele G (n = 14)	*p*-Value
Leukocytes, 10^9^/L	6.19 (4.40–8.34)	10.1 (6.74–14)	*p* = 0.023
Hematocrit, %	35 (30.2–37.4)	40.2 (35.2–43.5)	*p* = 0.040

^a^ Mann–Whitney test; statistically significant findings are denoted in bold.

**Table 7:** Impact of genetic variation on clinical course and laboratory parameters during Paxlovid treatment for hospitalized COVID-19 patients.

	**IFNAR2 rs2236757** Allele A (n = 15) Allele G (n = 19)	Admission	Discharge	*p*-Value ^a^
SpO_2_, %, median (IQR)	Allele A	96 (94–98)	98 (97–98)	*p* = 0.151
	Allele G	96 (92–97)	98 (97–98)	***p*** **= 0.019**
Segmented neutrophils, %	Allele A	55 (46–75)	66 (48–74)	*p* = 0.059
	Allele G	61 (47–70)	66 (52–78)	***p*** **= 0.029**
Eosinophils, %	Allele A	1 (0–2)	1 (0–1)	*p* = 0.169
	Allele G	1 (1–2)	1 (0–1)	***p*** **= 0.048**
ESR, mm/h	Allele A	7 (4–11)	5 (4–6)	***p*** **= 0.021**
	Allele G	5 (4–10)	4 (4–5)	*p* = 0.371
Platelet count, 10^9^/L	Allele A	173 (142–204)	215 (166–244)	***p*** **= 0.041**
	Allele G	193 (165–231)	220 (169–262)	*p* = 0.064
Hematocrit, %	Allele A	37.2 (34–44)	36.6 (30.8–41)	*p* = 0.132
	Allele G	40 (34.2–45)	37 (32–42.7)	***p*** **= 0.040**
APTT, s	Allele A	33.2 (29.4–35.3)	32.8 (24.6–35.8)	*p* = 0.177
	Allele G	33.2 (29.4–37)	29.8 (25–33.7)	***p*** **= 0.035**
Total bilirubin, mmol/L	Allele A	13.4 (11.1–19.1)	11.2 (10.7–14.1)	*p* = 0.128
	Allele G	13.7 (10.8–19.1)	11.2 (10.5–13.5)	***p*** **= 0.029**
AST, mmol/L	Allele A	23.3 (19–27.8)	25.5 (23.3–67.4)	*p* = 0.112
	Allele G	22.2 (16.6–25.8)	30.8 (23.3–94.1)	***p*** **= 0.014**
Creatinine, mmol/L	Allele A	103 (95–117)	92 (84–109)	***p*** **= 0.044**
	Allele G	96 (80–117)	98 (86–109)	*p* = 0.825
Albumin, g/L	Allele A	50 (45–57)	43 (37–51)	***p*** **= 0.023**
	Allele G	50 (45–56)	46 (42–51)	*p* = 0.159
	**ACE 2 rs2074192** Allele C (n = 22) Allele T (n = 8)	Admission	Discharge	*p*-Value ^a^
SpO_2_, %, median (IQR)	Allele C	96 (93.5–98)	98 (97–98)	***p*** **= 0.019**
	Allele T	96 (92–97.8)	97 (97–98)	*p* = 0.102
Segmented neutrophils, %	Allele C	57 (46–71.3)	66 (51.3–75.8)	***p*** **= 0.016**
	Allele T	57 (44.8–65.5)	68.5 (52.5–73.8)	*p* = 0.078
APTT, s	Allele C	33.3 (29.7–37.1)	29.8 (25.5–34.9)	***p*** **= 0.027**
	Allele T	32.8 (29.6–35.1)	29.8 (25.2–34.5)	*p* = 0.093
Fibrinogen, g/L	Allele C	3.99 (3.55–4.94)	3.33 (2.76–3.99)	***p*** **= 0.017**
	Allele T	3.63 (3.55–4.33)	3.83 (1.75–3.99)	*p* = 0.611
Total, mmol/L	Allele C	12.7 (10.8–15.9)	11.1 (10.6–13.7)	*p* = 0.112
	Allele T	12.9 (10.7–17.8)	10.8 (10.2–12)	***p*** **= 0.028**
AST, mmol/L	Allele C	22.4 (17.2–26.3)	32.3 (24.2–81.2)	***p*** **= 0.004**
	Allele T	21.9 (14.9–28.1)	29.1 (21.3–81.1)	*p* = 0.327
ALP, mmol/L	Allele C	140 (116–1600	122 (95.3–147)	*p* = 0.077
	Allele T	148 (125–165)	136 (94.3–148)	***p*** **= 0.025**
	**OAS3 rs10735079** Allele A (n = 20) Allele G (n = 13)	Admission	Discharge	*p*-Value ^a^
Segmented neutrophils, %	Allele A	61 (47.5–73.8)	70.5 (53.3–77.3)	***p*** **= 0.027**
	Allele G	55 (46–69.5)	63 (48.5–75)	*p* = 0.307
Eosinophils, %	Allele A	1 (0.25–2)	1 (0–1)	*p* = 0.134
	Allele G	1 (1–2.5)	1 (0–1)	***p*** **= 0.046**
Hematocrit, %	Allele A	38.6 (34.3–43.5)	37.2 (31.5–42)	***p*** **= 0.021**
	Allele G	42 (35.3–48.2)	41 (36.2–44)	*p* = 0.916
APTT, s	Allele A	34.2 (30–37.2)	29.8 (26.9–35.2)	***p*** **= 0.033**
	Allele G	33.4 (29.9–37.1)	29.1 (24.7–34.8)	*p* = 0.066
Fibrinogen, g/L	Allele A	3.99 (3.55–)5.05	3.63 (2.92–3.99)	*p* = 0.064
	Allele G	3.99 (3.55–4.1)	3.33 (2.11–3.88)	***p*** **= 0.031**
Total bilirubin, mmol/L	Allele A	13.2 (10.7–16.7)	11.1 (10.6–14.3)	*p* = 0.070
	Allele G	12.9 (11.1–20.6)	10.7 (10.3–13.2)	***p*** **= 0.021**
AST, mmol/L	Allele A	22.7 (19.4–27.3)	30.1 (24.5–67.4)	***p*** **= 0.006**
	Allele G	22.2 (18.6–26.8)	26.5 (23.9–96.3)	*p* = 0.116
ALP, mmol/L	Allele A	136 (115–152)	113 (93.8–144)	***p*** **= 0.025**
	Allele G	138 (121–156)	127 (94–151)	*p* = 0.196
	**OAS1 rs10774671** Allele A (n = 20) Allele G (n = 14)	Admission	Discharge	*p*-Value ^a^
Segmented neutrophils, %	Allele A	47.5 (61–73.8)	70.5 (53.3–77.3)	***p*** **= 0.027**
	Allele G	57 (46–69.3)	63 (48.8–75)	*p* = 0.183
Eosinophils, %	Allele A	1 (0.25–2)	1 (0–1)	*p* = 0.134
	Allele G	1 (1–2.25)	1 (0–1)	***p*** **= 0.032**
Hematocrit, %	Allele A	38.6 (34.3–43.5)	37.2 (31.5–42)	***p*** **= 0.021**
	Allele G	41 (35.8–47.7)	40.2 (35.2–43.5)	*p* = 0.638
Fibrinogen, g/L	Allele A	3.99 (3.55–55)	3.63 (2.92–3.99)	*p* = 0.064
	Allele G	3.99 (3.55–4.26)	3.33 (2.27–3.99)	***p*** **= 0.046**
Total bilirubin, mmol/L	Allele A	13.2 (10.7–16.7)	11.1 (10.6–14.3)	*p* = 0.170
	Allele G	13.3 (11.2–22)	10.9 (10.4–13.7)	***p*** **= 0.014**
AST, mmol/L	Allele A	22.7 (19.4–27.3)	30.1 (24.5–67.4)	***p*** **= 0.006**
	Allele G	22.4 (19.5–26.3)	26 (24.2–95.2)	*p* = 0.096
ALP, mmol/L	Allele A	136 (115–152)	113 (93.8–144)	***p*** **= 0.025**
	Allele G	137 (120–155)	125 (95–148)	*p* = 0.158

^a^ Wilcoxon matched pairs test; statistically significant findings are denoted in bold.

## Data Availability

Data are contained within the article.
